# MicroRNA and Transcriptomic Profiling Showed miRNA-Dependent Impairment of Systemic Regulation and Synthesis of Biomolecules in *Rag2* KO Mice

**DOI:** 10.3390/molecules23030527

**Published:** 2018-02-27

**Authors:** Abu Musa Md Talimur Reza, Yun-Jung Choi, Jin-Hoi Kim

**Affiliations:** Department of Stem Cell and Regenerative Biotechnology, Humanized Pig Research Centre (SRC), Konkuk University, Seoul 143-701, Korea; talimurku@konkuk.ac.kr (A.M.M.T.R.); yunjungc@konkuk.ac.kr (Y.-J.C.)

**Keywords:** *Rag2* knockout (KO) mouse, miRNA-dependent regulation, systemic impairments, biomolecules synthesis

## Abstract

The *Rag2* knockout (KO) mouse is a well-established immune-compromised animal model for biomedical research. A comparative study identified the deregulated expression of microRNAs (miRNAs) and messenger RNAs (mRNAs) in *Rag2* KO mice. However, the interaction between deregulated genes and miRNAs in the alteration of systemic (cardiac, renal, hepatic, nervous, and hematopoietic) regulations and the synthesis of biomolecules (such as l-tryptophan, serotonin, melatonin, dopamine, alcohol, noradrenaline, putrescine, and acetate) are unclear. In this study, we analyzed both miRNA and mRNA expression microarray data from *Rag2* KO and wild type mice to investigate the possible role of miRNAs in systemic regulation and biomolecule synthesis. A notable finding obtained from this analysis is that the upregulation of several genes which are target molecules of the downregulated miRNAs in *Rag2* KO mice, can potentially trigger the degradation of l-tryptophan, thereby leading to the systemic impairment and alteration of biomolecules synthesis as well as changes in behavioral patterns (such as stress and fear responses, and social recognition memory) in *Rag2* gene-depleted mice. These findings were either not observed or not explicitly described in other published *Rag2* KO transcriptome analyses. In conclusion, we have provided an indication of miRNA-dependent regulations of clinical and pathological conditions in cardiac, renal, hepatic, nervous, and hematopoietic systems in *Rag2* KO mice. These results may significantly contribute to the prediction of clinical disease caused by *Rag2* deficiency.

## 1. Introduction

The *Rag2* knockout (KO) mouse is one of the most important immune-compromised animal models used in preclinical studies and biomedical research [1]. *Rag2* KO mice have an impaired V(D)J recombination status. Normally the V(D)J recombination processes are regulated by a complex of *Rag2* and *Rag1* proteins, which can create DNA double-strand breaks at conserved recombination signal sequences, thus contributing to the development of both B- and T-lymphocytes [1]. Therefore, the depletion of *Rag2* gene can seriously impair the generation of B- and T-lymphocytes [2]. The *Rag2* KO mouse model is widely used for xenotransplantation experiments and preclinical studies of human diseases including cancers [3]. 

Studies have shown that the *Rag2* KO mice show alteration in stress and fear responses when compared with immune competent mice [4], and the T cells play important roles during these stress and fear responses by contributing to the learning and memory processes [4]. Another study showed that *Rag1* KO mice have impairments in social recognition memory [5], and the *Rag1* gene might have very important roles in the development and functioning of the central nervous system [5]. These evidences make it clear that, in addition to the impairment of the immune system, the immune-compromised status can alter many important signaling pathways involved in the systemic regulation, synthesis of biomolecules, and degradation processes. Considering that the metabolic pathways have important interactive roles with immune regulation process [6,7], it is important to investigate the possibility of alteration in systemic regulation and synthesis of biomolecules (such as l-tryptophan, serotonin, melatonin, dopamine, alcohol, noradrenaline, putrescine, and acetate) in *Rag2* KO mice, especially the molecules which are related to the learning and memory processes. For example, l-tryptophan is a precursor to the neurotransmitter serotonin and the hormone melatonin [8], and play a very important role in learning and memory [9], as well as in the development of fetus [10]. l-tryptophan is an essential non-polar aromatic α-amino acid [11], and requires a continuous supply through dietary medium for proper functioning of the biological processes [11,12] in humans and other animal species. Thus, imbalances in molecules like l-tryptophan could cause severe damages in biological and metabolic processes. 

Genomic and bioinformatics approaches are becoming increasingly popular to understand the physiological and immunological disorders of immune-compromised mouse strains [13]. However, the possible alteration in other systemic regulations (such as cardiac, renal, nervous, and hepatic systems) and synthesis of biomolecules including hormones in *Rag2* KO mice have not been clarified. MicroRNAs (miRNAs) as well as other non-coding RNAs are potent regulators of the expression of mammalian genes and are involved in various biologically important signaling pathways [14,15]. Therefore, specifying the involvement of regulatory miRNAs in these processes is also important to understand the physiological regulations in *Rag2* KO mice. Here, we have investigated the potential miRNAs involved in the regulation of pathways in systemic regulation and biomolecule degradation and synthesis. In this study, the altered target genes of deregulated miRNAs in *Rag2* KO mice are shown to be potentially involved in the morphological and physiological alteration of cardiac, renal, hepatic, and hematopoietic cells. Moreover, the degradation (l-tryptophan, melatonin, serotonin, dopamine, ethanol, noradrenaline, putrescine, and acetate) and biosynthesis (acetyl-CoA, uridine 5′-phosphate, palmitate, glycosaminoglycan-protein linkage region, and spermidine) of different biomolecules may be regulated by miRNAs. 

## 2. Material and Methods

### 2.1. Mice

Mouse strain lines were maintained on a congenic C57BL/6J background and were fed ad libitum with standard mouse chow purchased locally (Cargill Agri Purina, Inc., Seongnam-Si, Korea). Mice were supplied with abundant clean and pathogen-free drinking water. The *Rag2* knockout mice were maintained in a specific-pathogen-free (SPF) housing system. Experimental use of the animals was performed according to the guidelines of the Konkuk University Animal Care and Experimentation Community (IACUC approval number: KU12045). The *Rag2* knockout mouse was originally developed and was kindly provided by Taconic Biosciences, Inc. (Hudson, NY, USA). 

### 2.2. Tissue Collection

Three *Rag2* knockout (KO) and three wild type (WT) mice were sacrificed by cervical dislocation. The spleens were removed immediately by opening the abdominal cavity using a sterile scissor and forceps, and then stored in a marked vial at −80 °C for a short period of time until the tissues were used for isolation of RNA, as described below. 

### 2.3. RNA Isolation, Quality Check, and Microarray Analysis

Total mRNAs and miRNAs were isolated from spleen tissues of *Rag2* KO and WT mice using the miRNeasy mini kit (Qiagen, Valencia, CA, USA) according to the protocol recommended by the manufacturer. Concentration measurement and initial quality check of the RNAs was performed using a NanoDrop2000 spectrometer (Thermo Scientific, Waltham, MA, USA). Samples having an absorbance (260:280 nm) ratio around 1.8 was considered for further analysis. The quality of the isolated mRNAs and miRNAs was confirmed by detecting RNA integrity number (RIN) using a model 2100 Bioanalyzer (Agilent Technologies, Palo Alto, CA, USA). The miRNA expression microarray and gene expression microarray analyses were performed as described previously [1].

### 2.4. Selection of Differentially Expressed miRNAs and Genes for Downstream Analysis 

The miRNA data sets were GSM2750870, GSM2750871, and GSM2750872 for WT mice, and GSM2750867, GSM2750868, and GSM2750869 for *Rag2* KO mice [1]. The gene expression data sets were GSM2758547, GSM2758548 and GSM2758549 for the WT mouse, and GSM2758544, GSM2758545 and GSM2758546 for the *Rag2* KO mouse [1]. The miRNAs and genes which had at least a two-fold change in expression (upregulated or downregulated) in *Rag2* KO mice compared to WT mice were considered for downstream study. The miRNAs and genes which had less than two-fold changes in expression in *Rag2* KO mice compared to WT mice were excluded from this study to avoid the experimental errors derived from technical processes and individual variations. Further, miRNAs which did not show known targets (although they may have more than two-fold changes) were also excluded from this study. Concisely, only the miRNAs and genes which showed connection and had at least two-fold changes between *Rag2* KO and WT mice were considered for this study.

### 2.5. Prediction of Targets for Differentially Expressed miRNAs

The miRNAs which had at least two-fold changes in expression (upregulated or downregulated) in *Rag2* KO mice compared to WT mice were considered for target prediction. Putative target genes of these differentially expressed miRNAs between *Rag2* KO and WT mice were predicted using the miRsystem miRNA database (http://mirsystem.cgm.ntu.edu.tw/) using the search option “miRNAs to Target Genes”, which integrates data from seven miRNA-target prediction algorithm systems (DIANA, PITA, miRBridge, rna22, PicTar, miRanda, and TargetScan) and enables to find out the targets which are predicted by multiple algorithm systems. Prediction of targets by multiple algorithm systems strengthen the accuracy of targets prediction, and therefore, helps to find out target genes more precisely compared to the prediction using a single algorithm system. In this study, target genes predicted by at least three different programs (out of the seven mentioned above) were considered for downstream analysis. 

### 2.6. Generation of Heatmaps, miRNAs-Genes Interactinon Network and Catalytic Activity Networks 

Genes and miRNAs expression heatmaps, miRNAs-genes interaction network and catalytic activity networks for the selected deregulated miRNAs and genes were generated by uploading the data in the “Cytoscape software (http://www.cytoscape.org/) which is an open source platform to visualize molecular interaction networks and biological pathways as well as it could integrate these networks with different types of data including gene expression data, annotations or other [16]”. The genes and miRNAs expression heatmaps were generated by using “clusterMaker” which is a multi-algorithm clustering plugin for Cytoscape [17] with the commanding option for generation of “Hierarchical cluster”. The miRNAs-genes interaction network was generated by directly uploading the selected miRNAs name with their corresponding target genes to the Cytoscape software [16]. The catalytic activity network of the selected genes was generated by using another Cytoscape app known as “BiNGO” and is used to assess overrepresentation of gene ontology categories in biological networks [18]. Heatmaps and networks were exported from Cytoscape as image files for further use.

### 2.7. Ingenuity Pathway Analysis (IPA)-Based Canonical Pathways and Networks Detection 

IPA is a powerful web-based applications and analysis tool that utilize omics data (such as RNA-seq, microarrays, metabolomics, proteomics etc.) to interpret and identify new targets or candidate biomarkers within the context of biological systems [19]. IPA is widely used to accurately interpret omics data derived from life science research in a concise manner and to find out the corresponding canonical pathways and biological networks. Using omics data this software can identify upstream regulators, molecular and chemical interactions, cellular phenotypes and diseases (https://www.qiagen.com/sg/products/life-science-research/research-applications/gene-expression-analysis/analysis/ingenuity-pathway-analysis/#productdetails). In addition, IPA allows searches for targeted information on genes, proteins, chemicals, and drugs, and building of interactive models of experimental systems [19]. The software is supported by the Ingenuity Knowledge Base of a highly structured, detail-rich biological and chemical findings [19].

The differentially expressed genes between *Rag2* KO and WT mice which had opposite expression pattern to its targeting miRNA(s) were uploaded into the IPA software (Qiagen) along with the gene identifiers and respective expression values. The ‘core analysis’ was performed to analyze the canonical pathways, gene network, biological processes and upstream transcriptional regulator of the uploaded genes. The networks, canonical pathways and other visual presentations were automatically generated by the software.

### 2.8. Validation of Deregulated miRNAs and Expression of Genes by qRT-PCR

The deregulated expression levels of miRNAs were detected using quantitative real-time reverse transcription PCR (qRT-PCR) according to the instructions provided with the Mir-X miRNA qRT-PCR SYBR kit (Clontech Laboratories, Inc., Mountain View, CA, USA). For qRT-PCR analysis, specific sequences of miRNAs were regarded as miRNA-specific 5′ primers, and the mRQ 3′ primers provided with the kit were used as the 3′ primers for all miRNAs. The U6 RNA was used to normalize the threshold cycle (Ct) values and expression of miRNAs was quantified using the relative quantitation method (2^−ΔΔCt^). 

For the verification of dysregulated mRNA expression, cDNA was synthesized from the total RNA (extracted from different individuals than the microarray experiments) using the QuantiTect Reverse Transcription Kit (Cat No. 205313; Qiagen) according to the manufacturer’s protocol. Briefly, genomic DNA was eliminated by a reaction in the first step using gDNA wipeout buffer provided with the QuantiTect Reverse Transcription Kit and then cDNA was synthesized during the second step. The expression of selected genes in the *Rag2* KO and WT samples was detected by SensiFast SyBR Lo-ROX Kit (BIO-94003; Bioline, London, UK). Glyceraldehyde-3-phosphate dehydrogenase (GAPDH) was used to normalize the threshold cycle (Ct) values, and gene expression was quantified using the relative quantitation method (2^−ΔΔCt^). 

### 2.9. Cut-Off Line and Statistical Analysis

In case of microarray (both miRNA and mRNA) expression data, the cut off line was at least two-fold changes (upregulation or downregulation) in expression in *Rag2* KO mice compared to the WT mice. In case of qRT-PCR (both miRNA and mRNA) expression data, the relative expression (2^−ΔΔCt^ value) was determined for *Rag2* KO mice taking the WT mice as experimental control (standard), and U6 RNA (for miRNA) or GAPDH (for mRNA) were used to normalize the Ct values, while significance of differences between *Rag2* KO and WT mice (qRT-PCR genes and miRNAs expressions) was determined by the Student’s *t* test. Significance levels were determined as *p* < 0.05, *p *< 0.01, and *p* < 0.001, respectively. 

## 3. Results

Recently, it has become popular to revisit the correlative analysis of gene expression data using more recently generated data sets. In this study, we analyzed the data obtained from a recent comparative miRNA and mRNA microarrays in *Rag2* KO versus WT mice [1], and focused on the miRNAs that mediated systemic regulation and biomolecule metabolism in *Rag2* KO mice compared to the WT counterparts. To extend these data sets, we analyzed miRNA expression microarray data sets (GSM2750870, GSM2750871, and GSM2750872 for the WT mouse; GSM2750867, GSM2750868, and GSM2750869 for the *Rag2* KO mouse), as well as gene expression data sets (GSM2758547, GSM2758548 and GSM2758549 for the WT mouse, and GSM2758544, GSM2758545 and GSM2758546 for the *Rag2* KO mouse) [1] to investigate the altered systemic regulation and signaling during the synthesis of biomolecules, as well as assessing the involvement of regulatory miRNAs in these processes in *Rag2* KO mice. 

### 3.1. Deregulated miRNAs in Rag2 KO Mice Target Genes Involved in Systemic Regulation

**Figure 1:**
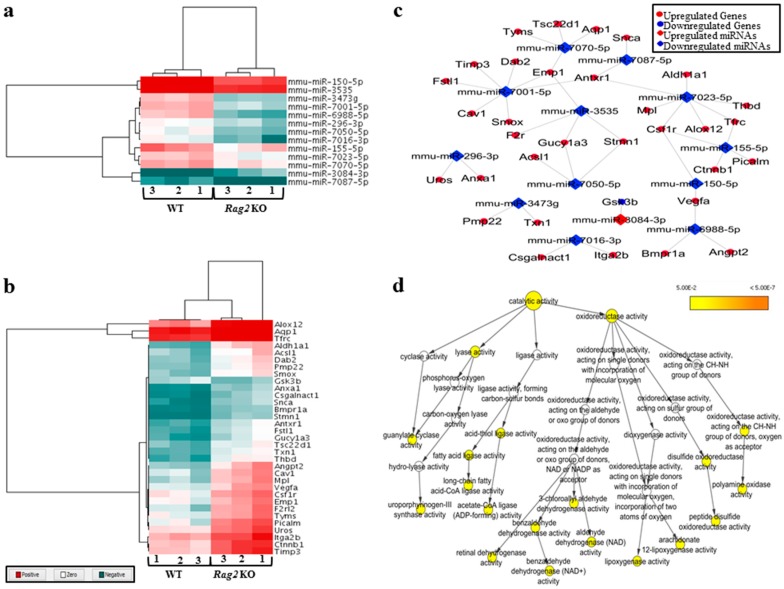
The figure showing deregulated miRNAs and their deregulated target genes in *Rag2* knockout (KO) mice having involvement in catalytic activity. (**a**) Heatmap of the deregulated miRNAs in *Rag2* KO mice which had deregulated targets; (**b**) heatmap of the deregulated target genes of the deregulated miRNAs in *Rag2* KO mice; (**c**) the interaction network showing the connection between deregulated miRNAs and deregulated target genes in *Rag2* KO mice; (**d**) the catalytic activity network showing the involvement of the deregulated target genes of the deregulated miRNAs in different catalytic processes.

**Figure 2:**
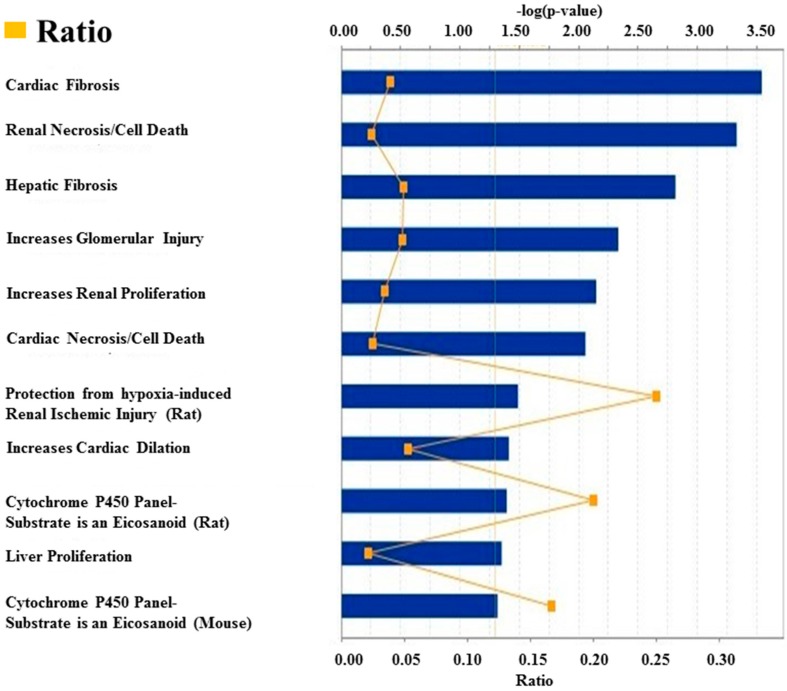
Ingenuity Pathway Analysis (IPA) toxicity list linking the experimental data with the potential clinical and pathological endpoints in *Rag2* KO mice. In this figure, the top potential clinical and pathological phenomena are listed. They are predicted to be present in *Rag2* KO mice, and include (but not limited to) cardiac abnormalities, renal necrosis/cell death, hepatic fibrosis and glomerular injury.

**Figure 3:**
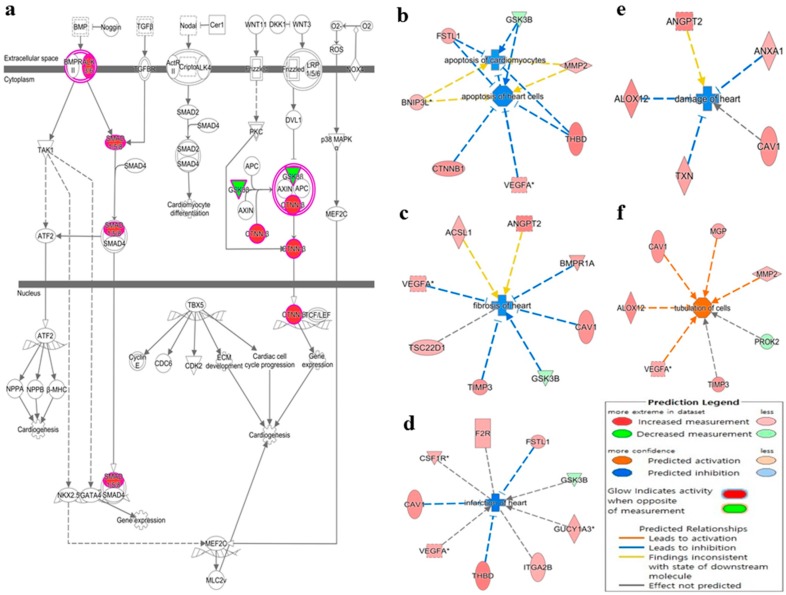
Potential alteration in cardiogenesis in *Rag2* KO mice. (**a**) The upregulated expression of factors indicating the promotion of cardiogenesis in *Rag2* KO mice; (**b**) apoptosis of the cardiomyocytes and heart cells are potentially inhibited in *Rag2* KO mice; (**c**) cardiac fibrosis is potentially inhibited in *Rag2* KO mice; (**d)** cardiac infection is potentially inhibited in *Rag2* KO mice; (**e**) the possibility of heart damages is potentially inhibited in *Rag2* KO mice; (**f**) cardiac tubulation is potentially upregulated in *Rag2* KO mice.

A group of deregulated miRNAs (Figure 1a) were identified as targeting molecules for a group of deregulated genes (Figure 1b) in the *Rag2* KO mice. The deregulated target genes of these miRNAs (Figure 1c) have regulatory involvement in the catalytic processes (Figure 1d). This indicates that the concerned miRNAs are potentially regulating many metabolic and systemic processes by an interacting relationship with the respective target genes. These upregulated gene expressions were potentially involved in the processes for systemic regulations and diseases, such as hepatic fibrosis, renal necrosis, and regulation of renal, cardiac, and liver cell proliferation (Figure 2). The processes involved during cardiogenesis were enhanced in the *Rag2* KO mice. Factors involved in the promotion of cardiogenesis pathways were upregulated (Figure 3a), whereas apoptosis of the cardiomyocytes and heart cells were inhibited (Figure 3b). Furthermore, fibrosis, infection, damage, and tubulation of heart cells (Figure 3c–f, respectively) might be regulated by the concerned gene sets. These predicted results indicated that the depletion of *Rag2* gene could cause cardiac injuries in a manner that is dependent on miRNA.

Similarly, the *Rag2* KO mice might be vulnerable to other diseases, such as organismal injury and abnormalities including cancer and gliosis (Supplementary Figure S1), along with renal dysfunction (Supplementary Figure S2). For example, the apoptosis and deformities of kidney cells were predictably activated in *Rag2* KO mice (Supplementary Figure S2a–d). In contrast, the proliferation and regeneration ability of the liver cells were potentially upregulated in the *Rag2* KO mice (Supplementary Figure S2e,f). Similarly, the generation of blood cells and blood coagulation factors were predictably elevated (Supplementary Figure S2g,h). However, the bleeding time was potentially decreased (Supplementary Figure S2i) in the *Rag2* KO mice. In addition, the factors involved in the survival and coagulation of cells were upregulated in the *Rag2* KO mice (Supplementary Figure S2j,k). Taken together, these findings indicate an alteration during the systemic regulation (especially cardiac, hepatic, renal, and hematopoietic regulation) of *Rag2* KO mice compared to the WT counterparts.

### 3.2. Deregulated miRNAs in Rag2 KO Mice Target Genes Involved in Biomolecule Synthesis 

**Figure 4:**
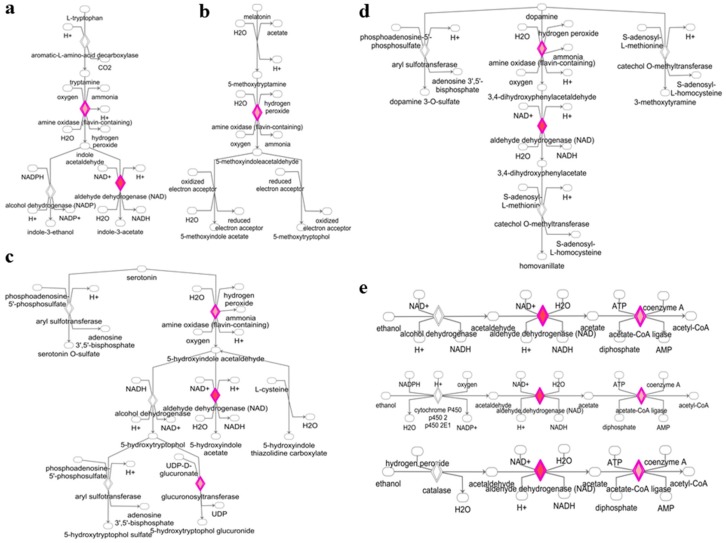
Alteration of biomolecule synthesis and degradation pathways in *Rag2* KO mice. (**a**) Upregulation of the l-tryptophan degradation signaling; (**b**) upregulation of melatonin degradation signaling; (**c**) upregulation of the serotonin degradation signaling; (**d**) upregulation of the dopamine degradation signaling; (**e**) upregulation of the ethanol to acetyl-coA conversion signaling.

**Figure 5:**
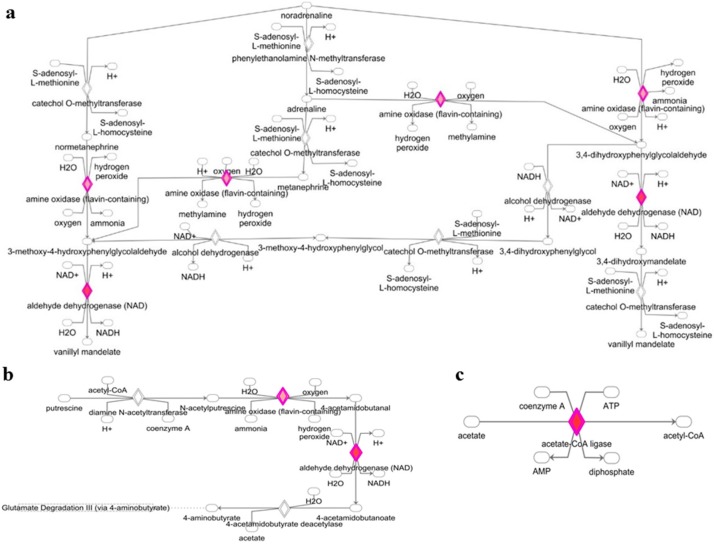
Alteration of biomolecule synthesis and degradation pathways in *Rag2* KO mice. (**a**) Upregulation of the noradrenaline degradation signaling; (**b**) upregulation of the putrescine degradation pathway; (**c**) upregulation of the acetate to acetyl-coA conversion signaling.

The genes targeted by deregulated miRNAs are also involved in the degradation and biosynthesis pathways of different biomolecules. As shown in Figure 4 and Figure 5, targeted gene expressions of deregulated miRNAs were identified in the l-tryptophan degradation pathway (Figure 4a), melatonin degradation pathway (Figure 4b), serotonin degradation pathway (Figure 4c), dopamine degradation pathway (Figure 4d), alcohol degradation pathway (Figure 4e), noradrenaline degradation pathway (Figure 5a), putrescine degradation pathway (Figure 5b), and acetate to acetyl-CoA conversion pathway (Figure 5c). These data indicated the potentially important roles of these miRNAs in the regulation of small molecules and hormonal activity in the *Rag2* KO mice. Similarly, target gene expressions of the deregulated miRNAs were also involved in the regulation of pathways related to the metabolism and conversion of some other molecules, such as uridine 5′-phosphate, palmitate, glycosaminoglycan-protein, and l-ornithine to putrescine conversion (Supplementary Figure S3). Thus, the biosynthesis and degradation of different biomolecules in *Rag2* KO mice are potentially altered in a miRNA-dependent manner. 

### 3.3. Experimental Validation 

**Figure 6:**
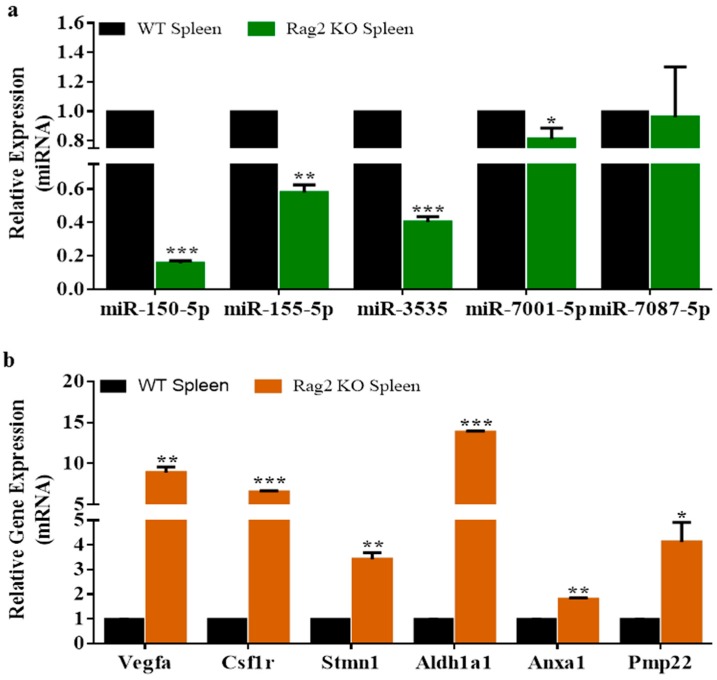
Validation of the deregulated miRNAs and genes expression in *Rag2* KO mice. (**a**) Validation of the downregulated expression of miR-150-5p, miR-155-5p, miR-3535, miR-7001 and miR-7087-5p in *Rag2* KO mice; (**b**) validation of the upregulated expression of the corresponding targets (*Vegfa*, *Csf1r*, *Stmn1*, *Aldh1a1*, *Anxa1* and *Pmp22*) of the downregulated miRNAs in *Rag2* KO mice. *** *p* < 0.001, ** *p* < 0.01 and * *p* < 0.05.

Downregulated miRNAs were identified as potentially involved in the regulation of upregulated gene expressions in *Rag2* KO mice (Figure 1), which are the potential regulators of systemic processes (Figure 2 and Figure 3), and the synthesis and degradation of biomolecules (Figure 4 and Figure 5). For example, *Vegfa*, *Csf1r*, *Aldh1a1*, *Pmp22*, *Anxa1*, *Stmn1*, and others are involved in the regulation of different pathways related to the systemic regulation (cardiac, renal, hepatic, and others), synthesis of biomolecules, and degradation (putrescine, melatonin, serotonin, dopamine, and others). The interdependent relationship between deregulated miRNAs and gene expressions was further confirmed by the qRT-PCR miRNA and gene expressions data (Figure 6). 

## 4. Discussion

Many biological and molecular events are involved in the bio-physiological processes of healthy and diseased individuals [20,21,22]. Deregulation in any of the participating molecules causes imbalances in others and potentially triggers the dysfunction of the entire process of systemic and functional regulation of bio-physiological events [20,21,22]. The deletion of *Rag2* gene causes immune deficiency in mice, and plays a role in the deregulation of many other important genes and miRNAs that are involved in other physiological processes [1]. As shown in this study, the molecules involved in the degradation of several important hormones and biomolecules (such as l-tryptophan, serotonin, melatonin, dopamine, alcohol, noradrenaline, putrescine, and acetate) were upregulated in *Rag2* KO mice, while the miRNAs targeting for that particular gene were downregulated. The findings suggest the potential miRNA-dependent regulation of these physiological events. For example, l-tryptophan is an essential amino acid in humans [23]; the human body cannot synthesize tryptophan and it must be supplemented through the diet. Tryptophan is a building block of many proteins and plays important structural and functional roles [23]. Moreover, tryptophan is a precursor of the neurotransmitter serotonin and the hormone melatonin [8,24]. Hence, the hydroxylases of l-tryptophan leads to the synthesis of serotonin [25,26]. Also, melatonin is synthesized from serotonin by the catalytic activity of *N*-acetyltransferase and 5-hydroxyindole-*O*-methyltransferase [27]. This means that the balanced availability of l-tryptophan is a prerequisite for the synthesis of both serotonin and melatonin. Studies have shown that both *Rag2* KO and *Rag1* KO mice show alteration in behavioral patterns [4,5], especially in the stress and fear responses [4] and impairments in social recognition memory [5], the *Rag2* and *Rag1* genes play roles mainly by contributing to the learning and memory processes [4] along with the development and functioning of the central nervous system [5]. l-tryptophan is a precursor to the neurotransmitter serotonin and the hormone melatonin [8], and plays a very important role in learning and memory [9], and in the development of the fetus [10]. Therefore, the rapid degradation of l-tryptophan could be the ultimate reason for the potential degradation of serotonin and melatonin biosynthesis following the impairments of behavioral patterns including stress and fear responses or social recognition memory and nervous system development in *Rag2* KO mice. 

l-Tryptophan also plays important roles in the biosynthesis of many other micro-molecules that are important for the functional and structural maintenance of biological systems [28,29,30,31,32]. For example, indole and its derivatives are bioactive compounds synthesized from l-tryptophan by gut microflora that produce tryptophanase [28,29], while indole and its derivatives play important neuroprotective antioxidant roles and prevent neural diseases and disorders [28,29]. Moreover, indole is also associated with vascular and renal diseases; indoxyl sulfate, a metabolic product of indole, is toxic in high concentrations and contributes to vascular diseases including increased oxidative stress, proliferation of smooth muscle cells, and thickness of aortic wall, as well as increased calcification and renal dysfunction [28]. As shown in this study, the *Rag2* KO mice potentially have altered cardiac signaling (Figure 3) and deformed renal cells (Supplementary Figure S2). Therefore, it might be concluded that the degradation of l-tryptophan could cause severe dysfunction in neural, vascular, metabolic, and renal systems. Also, the imbalances in the systemic regulation, biomolecules synthesis and behavioral patterns of the *Rag2* KO and *Rag1* KO mice might be the result of the deregulation of the miRNA-dependent l-tryptophan metabolism pathways. Thus, the depletion of *Rag2* gene could also cause impairments in the nervous, cardiac, renal, hepatic, and hematopoietic regulations in a miRNA-dependent manner. Finally, our observations may provide significant clues to developing new drugs to cure clinical symptoms caused in *Rag2/Rag1*-deficient mice. However, functional validation studies are required, such as *Luciferase* activity assay or particular miRNA knockout/knockdown cell or animal model studies to further confirm the roles of these miRNAs in systemic regulation and biomolecules synthesis.

## Supplementary Materials

The supplementary materials are available online.
