# Supplementary material for: MicroRNA and Transcriptomic Profiling Showed miRNA-Dependent Impairment of Systemic Regulation and Synthesis of Biomolecules in Rag2 KO Mice
Source: Molecules. 2018 Feb 27;23(3):527. doi: 10.3390/molecules23030527 (PMC6017002; doi:10.3390/molecules23030527)
Supplement: Supplementary file 1 [file molecules-23-00527-s001.pdf]

molecules

MDPI

Supplementary Information

MicroRNA and transcriptomic profiling showed miRNA-dependent impairment of systemic regulation and synthesis of biomolecules in *Rag2* KO mice

Abu Musa Md Talimur Reza, Yun-Jung Choi, and Jin-Hoi Kim *

Department of Stem Cell and Regenerative Biotechnology, Humanized Pig Research Centre (SRC), Konkuk University, Seoul 143-701, Republic of Korea; talimurku@konkuk.ac.kr (A.M.M.T.R.); yunjungc@konkuk.ac.kr (Y.-J.C.)

* Correspondence: jhkim541@konkuk.ac.kr; Tel: +82-2-450-3687; Fax: +82-2-458-5414

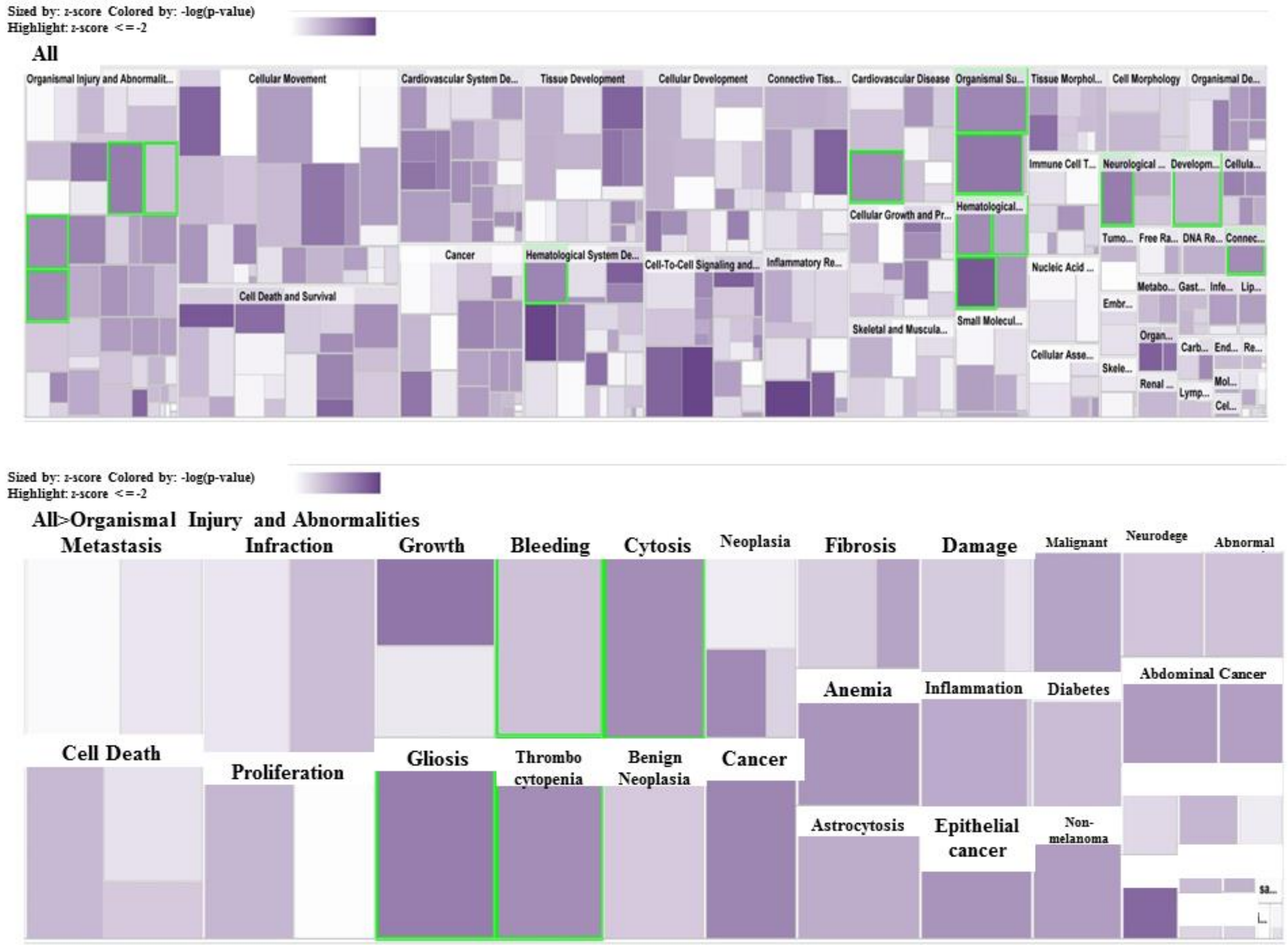

Supplementary Figure S1. Heatmap showing the alteration status in the physiological and systemic processes of *Rag2* KO mice. (**a**) Heatmap showing the alteration status of all biological and physiological processes in *Rag2* KO mice. (**b**) Heatmap showing the alteration status in the 'Organismal Injury and Abnormalities' in *Rag2* KO mice. Sized by: z-score, Colored by: −log(p-value), Highlight: z-score <= −2.

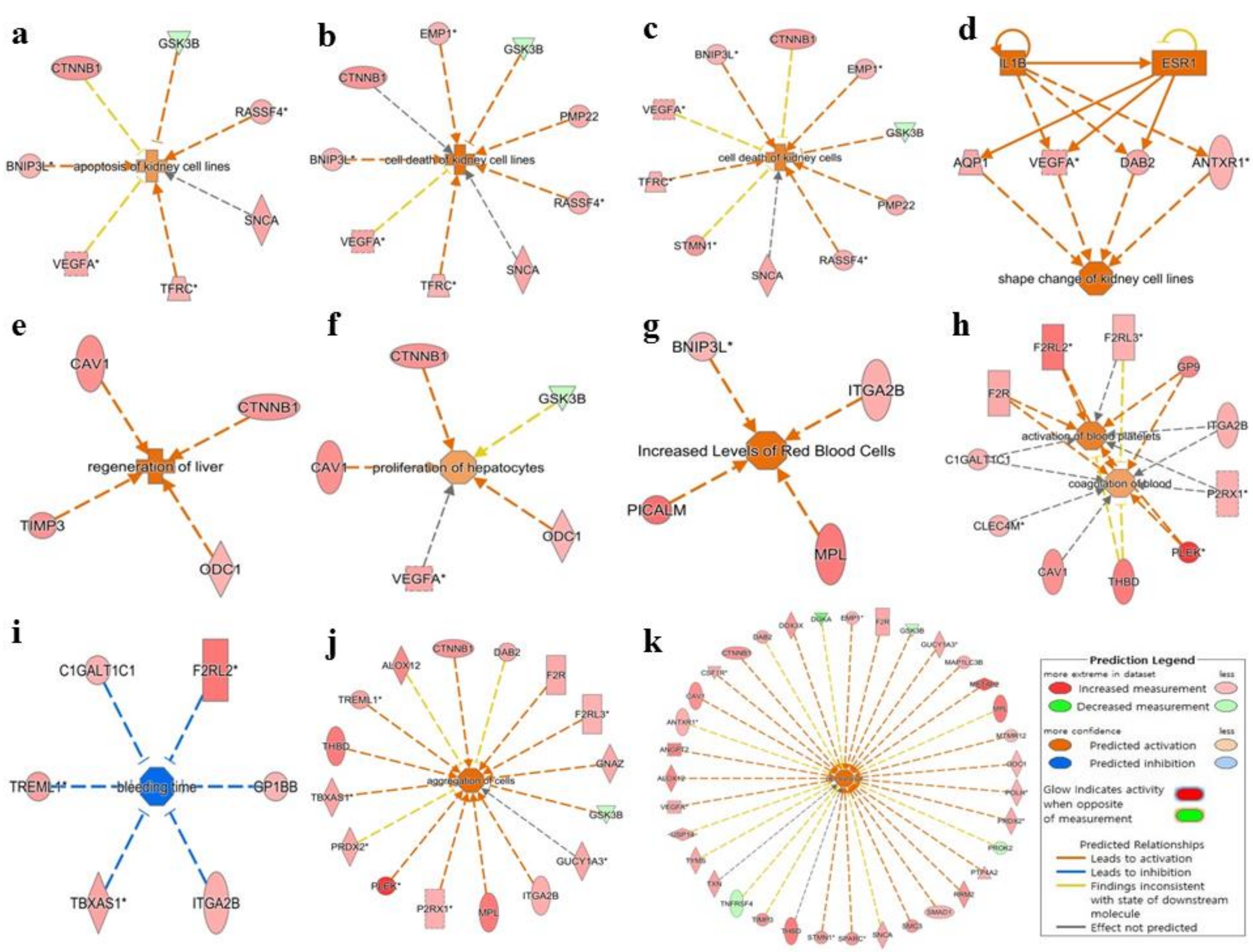

Supplementary Figure S2. Alteration status in different systemic regulations in *Rag2* KO mice. (**a–c**) Upregulated signaling for apoptosis of kidney cells. (**d**) Signaling for deformities in kidney cells upregulated. (**e–f**) Increased regeneration and proliferation of hepatic cell increases. (**g–h)** Signaling related to the formation of blood cells is activated. (**i**) Bleeding time in *Rag2* KO mice is potentially inhibited. (**j**) Cell aggregation signaling is upregulated. (**k**) Cell survival signaling is upregulated.

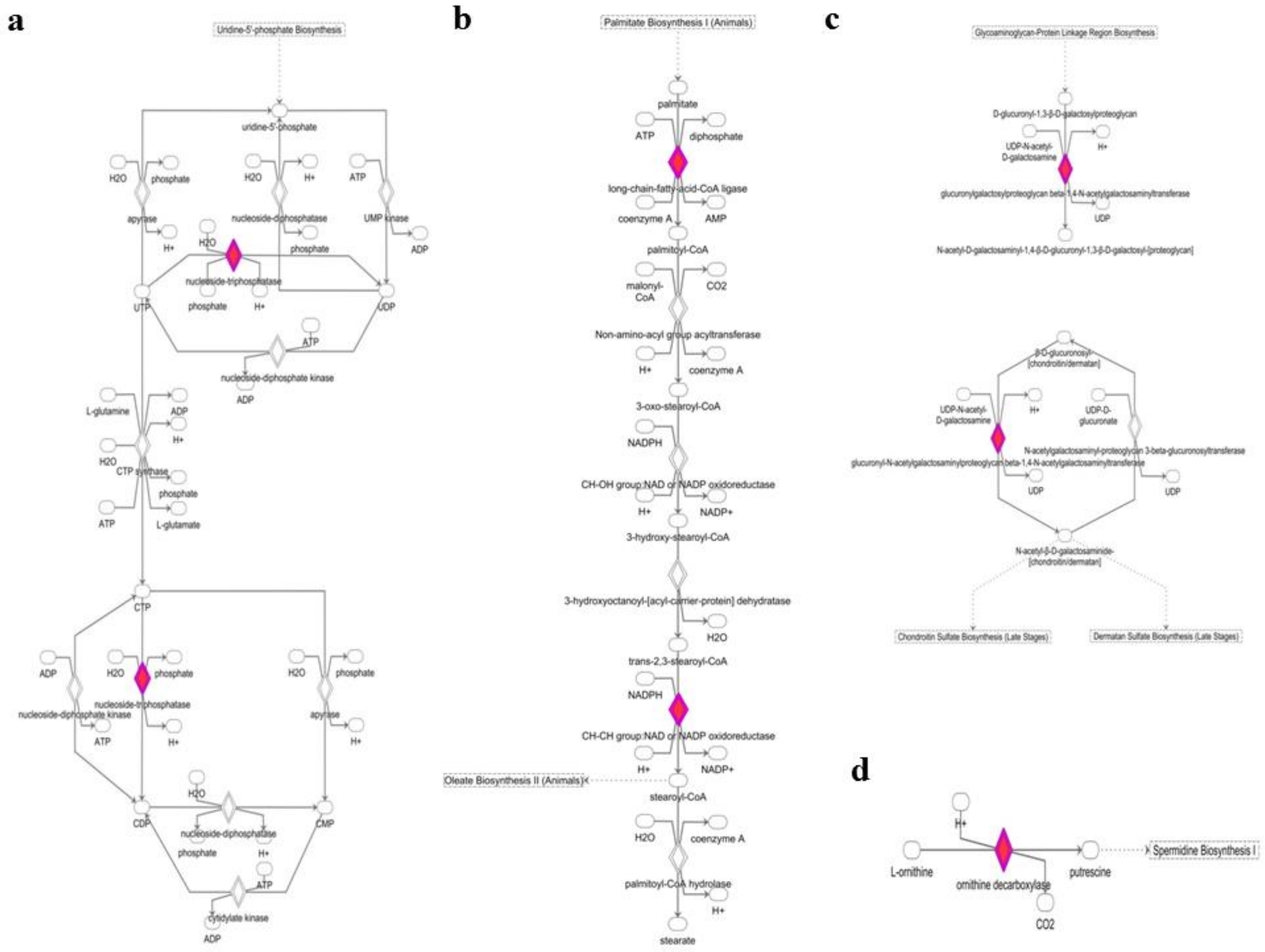

Supplementary Figure S3. Alteration in signaling related to the metabolism and conversion of biomolecules in *Rag2* KO mice (**a**) uridine 5′-phosphate; (**b**) palmitate; (**c**) glycosaminoglycan-protein; (**d**) L-ornithine to putrescine conversion.
